# Review of Plant Extracts and Active Components: Mechanisms of Action for the Treatment of Obesity-Induced Cognitive Impairment

**DOI:** 10.3390/brainsci13060929

**Published:** 2023-06-08

**Authors:** Ike de la Peña, Timothy Afable, Vina Rose Dahilig-Talan, Philip Cruz

**Affiliations:** 1 Department of Pharmaceutical and Administrative Sciences, School of Pharmacy, Loma Linda University, Loma Linda, CA 92350, USA; tafable@llu.edu; 2 School of Pharmacy, Manila Adventist College, Pasay City 1300, Philippines; vinarosedahilig@yahoo.com; 3 Herbanext Laboratories, Inc., Negros South Road, Bago City 6101, Philippines; philipcruz.herbanext@gmail.com

**Keywords:** obesity, cognitive impairment, plants, plant-derived compounds, herbal medicine

## Abstract

Obesity has been shown to negatively impact cognitive functions, but effective treatments for obesity-induced cognitive impairment are lacking. Natural dietary and plant products, functional foods, and plant-derived compounds have gained attention as potential remedies in part due to the nootropic properties of plants and certain plant-derived agents. This review discusses plant extracts and plant-derived substances that have been shown to ameliorate obesity-induced cognitive impairment in animal models. Mechanistic evaluations of their therapeutic effects are also summarized. A literature search was conducted using PubMed and Google Scholar databases, resulting in the review of 27 English language articles meeting the inclusion criteria. The nine plants (e.g., Ashwagandha, Adzuki bean, and olive) and 18 plant-derived substances (e.g., curcumin, Huperzine A, and Roxburgh’s jewel orchid polysaccharides) included in this review improved obesity-induced cognitive impairment through several mechanisms, including attenuation of neuroinflammation, improvement in both central and peripheral insulin resistance, enhancement of neuroprotection and neurogenesis, and modulation of the synthesis and release of cognition-associated neurotransmitters. Based on these findings, plants and plant-derived substances may hold promise for the prevention and treatment of obesity-induced cognitive impairment. Further research is warranted to explore the clinical potential of these plant-derived treatments and to elucidate their underlying molecular mechanisms.

## 1. Introduction

Obesity has become a global pandemic, affecting billions of people worldwide [1]. It poses a serious public health issue due to its association with increased risks of many diseases, including diabetes, cardiovascular and musculoskeletal diseases, cancer, and mental health disorders [2]. Furthermore, it has been linked to neurological and neurodegenerative disorders [3,4,5], with cognitive dysfunction or impairment receiving significant attention in recent years [6]. Indeed, a recent systematic review and meta-analysis of 16 studies involving 5,060,687 participants reported a significant association between high waist circumference, a measure of central obesity, and heightened risk of cognitive impairment and dementia [7]. Another meta-analysis comprising 29 longitudinal cohort studies involving 20,083 individuals revealed an elevated risk of developing dementia in individuals with a high body mass index (BMI) (BMI > 29 kg/m^2^) during mid-life [8]. With an aging population, the financial and healthcare burdens of cognitive impairment, and the lack of curative treatments for dementia and Alzheimer’s disease (AD) [9], it is crucial to address the increasing prevalence of obesity and develop treatments that specifically target its cognitive outcomes.

Prevention and treatment of obesity may help improve obesity-induced cognitive dysfunction. Alternatively, addressing cognitive impairment may help improve dietary self-regulatory abilities, which may lead to better food and lifestyle choices and sustainable weight loss in obese individuals [10,11]. Unfortunately, therapeutic interventions targeting obesity-linked cognitive dysfunction are currently lacking. However, recent studies have emerged reporting cognitive-improving effects of natural dietary and plant products, functional foods, and plant-derived components in preclinical models of obesity. The growing interest in plants and natural substances has been fueled by the need to develop safer alternative or complementary treatments for obesity and obesity-induced mental outcomes, the lack of curative treatments for cognitive impairment, and emerging attention to the nootropic properties of plants and plant-derived agents such as phenolics and flavonoids [12,13], which could be useful in treating obesity-induced cognitive impairment. This review provides a summary of plant extracts and plant-derived substances that have been demonstrated to alleviate obesity-induced cognitive impairment. It also examines the results of mechanistic evaluations of their cognitive-enhancing effects. The purpose of this paper is to shed light on the pathological mechanisms of obesity-induced cognitive impairment and to provide information that supports the potential utility of plant-based and herbal-derived compounds in ameliorating obesity-induced cognitive impairment.

## 2. Methods

The manuscript includes research articles gathered from PubMed that examined the effectiveness of plants and plant-derived substances in improving obesity-induced cognitive impairment. A hand search of relevant articles was also performed using Google Scholar. The search strategy employed included combining these keywords: “natural products”, “plant”, “plant-derived compound”, “plant extract”, “flavonoid”, “phenolic”, “obesity”, “cognitive dysfunction”, or “cognitive impairment”. There was no date limit applied in the search to ensure a comprehensive review of the available literature. The final database search was run in March 2023. This process yielded 339 papers covering a wide range of research article types. To focus specifically on plants and plant-derived compounds evaluated in animal models of obesity, only preclinical studies were included, while other animal models, such as mouse models of AD and type 2 diabetes, were excluded. Editorials, reviews, commentaries, and case reports were also excluded from this review. Furthermore, non-plant sources (e.g., mushrooms) and semi-synthetic compounds (i.e., bardoxolone methyl, Bis 3) were excluded based on predefined criteria. In total, 27 English language articles meeting the inclusion criteria were reviewed for this paper. The selected articles spanned from 2013 to 2022, ensuring comprehensive coverage of the relevant literature. Notably, 70% of the selected articles were published in the last five years.

## 3. Obesity-Induced Cognitive Impairment: Role of Hippocampus and Prefrontal Cortex

The hippocampus, a brain region responsible for regulating cognition and memory, is commonly implicated in obesity-induced cognitive impairment. Studies involving humans reported associations between higher BMI (>30 kg/m^2^) and reductions in grey matter volume and white matter integrity in the hippocampus and other brain areas [14,15,16], highlighting the detrimental effects of obesity on brain structure [17]. Moreover, functional magnetic resonance imaging (fMRI) studies reported a reduction in functional activity in the hippocampus in obese individuals performing an episodic memory task [18], further supporting the link between obesity and cognitive impairment. Preclinical studies have also shown the impaired performance of obese animals in behavioral tasks that require an intact hippocampus (e.g., Morris water maze test), accompanied by pro-inflammatory gene expression in this brain area. Additionally, other studies have shown altered hippocampal functions in obese individuals that affect their valuation of food and hinder their attempt to lose weight [19,20].

Obese individuals also show impairment in executive functions or higher cognitive processes that enable forethought and goal-directed actions [21], implying a key role of the prefrontal cortex (PFC) in obesity-induced cognitive impairment. Neuroimaging studies have shown hypoactivation in the PFC of obese individuals during the performance of executive function tasks (e.g., go/no-go), as well as altered connectivity, structural abnormalities, and task-related PFC dysfunction in obese humans [22]. These studies also reported grey matter atrophy within the parietal, temporal, and frontal lobes, particularly the PFC, in obese individuals [23]. Preclinical studies have also shown that obese rats perform poorly on cognitive tasks that require an intact PFC, such as Y-maze and T-maze tests [3]. Thus, obesity may cause structural and functional changes within the PFC that eventually result in altered cognitive functions in obese individuals [10]. It is important to note, however, that poor diet can also have direct effects on cognitive performance and age-related cognitive decline [24], independent of weight gain and obesity.

## 4. Putative Mechanisms of Obesity-Induced Cognitive Impairment

**Figure 1:**
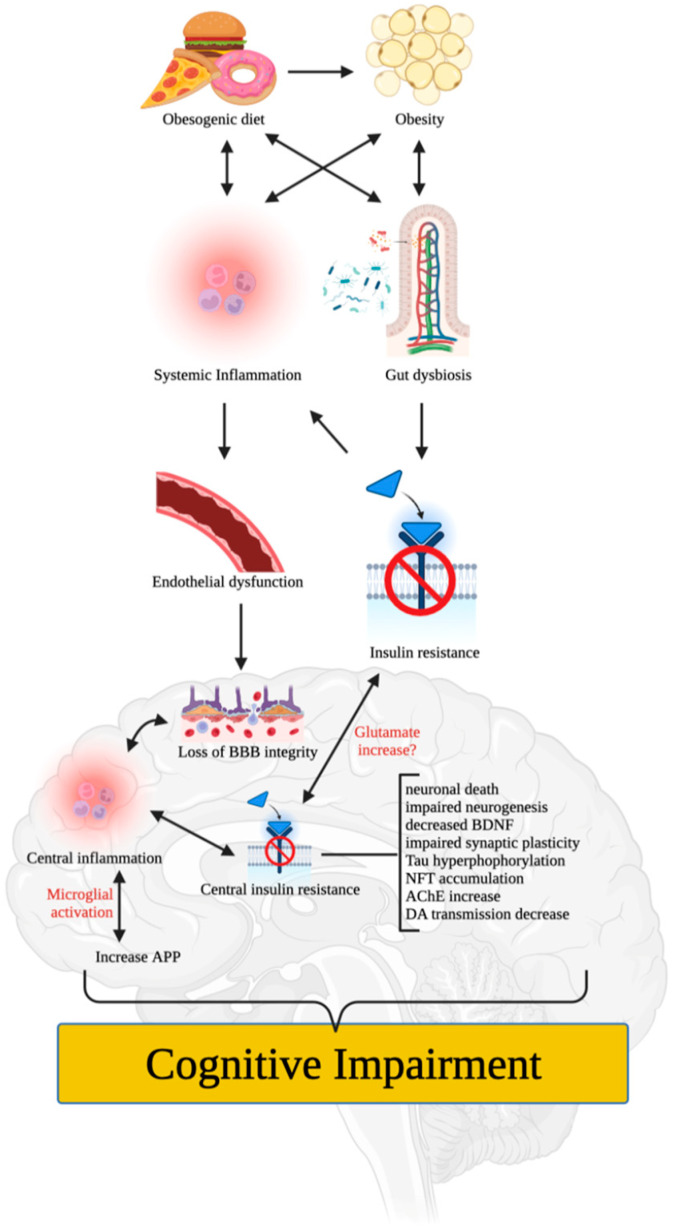
Mechanisms by which poor diet and obesity impair cognitive function. Poor diet and/or obesity induce low-grade systemic inflammation that damages the blood-brain barrier and induces central inflammation, microglial activation, and pro-inflammatory protein expression, resulting in neuronal death, impaired neurogenesis, and synaptic remodeling. Obesity-induced alterations in the gut microbiome, metabolic dysfunction, insulin resistance, and white adipose tissue expansion also contribute to cognitive impairment. Disruption of neurotransmitter systems, such as cholinergic, dopaminergic, and glutamatergic systems, leads to reductions in acetylcholine and dopamine levels and dysfunction in glutamate signaling, which further affects memory, learning, and cognition, leading to cognitive deficits. Figure created with BioRender.com (accessed on 17 May 2023).

Numerous mechanisms have been proposed to explain how poor diet and/or obesity can impair cognitive function. For instance, poor (i.e., obesogenic) diet and/or obesity can induce low-grade systemic inflammation that impairs the integrity of vascular components (e.g., endothelial cells and tight junction proteins) [25] (Figure 1). This can lead to loss of blood-brain barrier (BBB) integrity, central inflammation, microglial infiltration, activation, and proliferation, and induction of pro-inflammatory nuclear factor-kappa B (NF-κB) that increases the expression of pro-inflammatory proteins such as interleukin 1 beta (IL-1β), tumor necrosis factor-alpha (TNFα), and interleukin 6 (IL-6) [6,26,27]. These effects can accelerate neuronal death, impair neurogenesis, and produce synaptic remodeling that affects hippocampal and/or PFC functions, leading to cognitive dysfunction or impairment [27].

Moreover, poor diet and/or obesity can alter the gut microbiome and promote white adipose tissue expansion and metabolic dysfunction such as insulin resistance, which may impair cognitive function independently, and through systemic inflammation [28]. It is worth noting that neuroinflammation can promote central insulin resistance and vice versa [29,30], rendering neuronal cells more susceptible to metabolic and oxidative stress, which can alter neurogenesis (e.g., decreased expression of brain-derived neurotrophic factor [BDNF]), synaptic plasticity, memory capacity, and cognitive performance. Disruption of the insulin signaling may also lead to over-phosphorylation of the Tau protein, resulting in the accumulation of neurofibrillary tangles (NFTs) that induce neuronal apoptosis, as seen in AD [31]. Recent studies have also shown that central inflammation can increase the amyloid precursor protein (APP) [32], which plays a significant role in synaptic formation, neuronal plasticity, and iron export and is involved in the pathophysiology of AD.

Cognition, learning, and memory heavily rely on neurotransmitter systems such as cholinergic, dopaminergic, and glutamatergic systems. Obesity and/or poor diet may lead to cognitive dysfunction via insulin resistance, which has been shown to increase acetylcholinesterase (AChE) activity [33] and reduce synaptic levels of acetylcholine (ACh). Furthermore, significant reductions in dopamine have been observed in the brains of both obese humans and animals [10,34]. Prolonged consumption of an obesogenic diet was reported to damage the brain’s dopamine network and impair learning and memory in animals [35,36], indicating the critical role of dopamine in obesity and/or poor diet-induced cognitive deficits [11]. Glutamate, the most dominant excitatory neurotransmitter in the brain, is also involved in memory, learning, and cognition. Labban et al. [37] found increased levels of glutamate in brain tissues of high-fat diet (HFD)-fed obese rats, in agreement with previous findings of a focally extended excitatory postsynaptic current in HFD-fed mice, ascribed to lowered glutamate buffering and/or blunted glutamate (i.e., N-methyl-D-aspartate receptor [NMDA]) receptors with higher glutamate signaling in the brain [38]. These changes were proposed to cause dysfunction in extrasynaptic NMDA receptors, a reduction in long-term potentiation, and abnormalities in mitochondrial functions [37].

## 5. Plants Investigated for Their Potential to Ameliorate Obesity-Induced Cognitive Impairment

**Figure 2:**
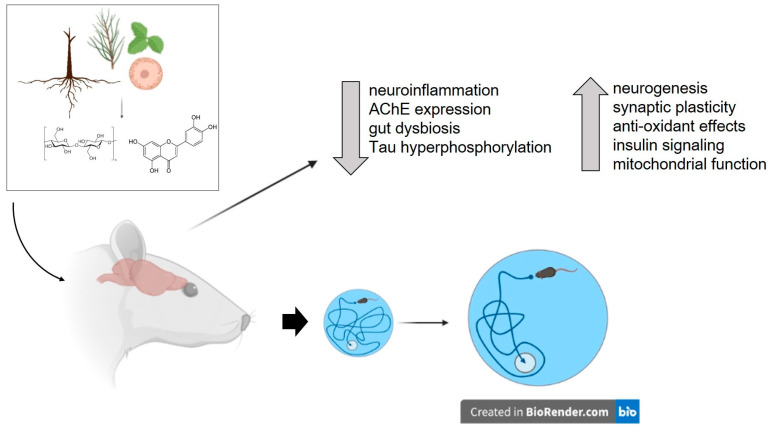
Potential therapeutic effects of plants and plant-derived compounds on obesity-induced cognitive impairment. Various mechanisms through which plants and isolated compounds can improve cognitive function in obese individuals include attenuation of neuroinflammation, reduction in acetylcholinesterase (AChE) expression, improvement of central and peripheral insulin resistance, enhancement of neurogenesis and neuroprotection, and modulation of the synthesis and release of cognition-associated neurotransmitters. The figure also depicts the improvement of spatial cognition, as measured by the Morris water maze, in an obese rat supplemented with plant or plant-derived compounds. Figure created with BioRender.com (accessed on 17 May 2023).

The use of herbal remedies and plant products as therapeutic agents has a long history in many cultures and has been instrumental in discovering drugs for various therapeutic areas. The natural origin of plant substances and their minimal adverse effects have led to an increase in their use as nootropic agents, including for AD [12,13]. Recently, several studies have explored the potential therapeutic role of plants and plant-derived substances in treating obesity-induced cognitive impairment (Table 1 and Table 2). Of note, the efficacy of these herbs and their components have been examined using cognitive tests such as the Morris water maze, T maze, Y-maze, and novel object recognition tests. Additionally, most of these studies have also examined potential mechanisms underlying the therapeutic efficacy of these plants. The results of these studies indicate that plants and plant-derived compounds improve obesity- or high-fat/high-sucrose diet-induced cognitive impairment through various mechanisms, including attenuation of neuroinflammation, improving central and peripheral insulin resistance, enhancing neurogenesis and neuroprotection, and affecting the synthesis and release of cognition-associated neurotransmitters (Figure 2). The following section will discuss the key details about plants, their cognitive-enhancing effects, and the findings from studies investigating their mechanism(s) of action.

### 5.1. Ashwagandha (Withania somnifera)

Ashwagandha (ASH), an important herb in Ayurvedic medicine, displays various pharmacological activities and therapeutic potential against several types of cardiovascular comorbidities, hyperlipidemia, and obesity [48]. The memory-enhancing effect of the plant was also shown in previous studies, for instance, in animal models of stroke and AD [49,50]. In a previous study, obese female rats given the dry leaf powder of ASH (1 mg/g of body weight for 12 weeks) showed improvement in working memory as evaluated by the novel object recognition test (*p* ≤ 0.05) [39]. ASH restored BDNF levels and tropomyosin receptor kinase B (TRKB) expression and increased the expression of synaptic plasticity and cell survival markers, such as the neural cell adhesion molecule (NCAM) and calcium/calmodulin-dependent protein kinase II alpha (CaMKIIα) in both hippocampus and the piriform cortex of these animals. ASH also enhanced the mRNA expression of phosphatidylinositol 3-kinase/protein kinase B (PI3K/AKT), whose signaling pathway prevents cellular apoptosis and promotes cell survival. Notably, the extract did not affect the body weight and blood glucose levels of obese animals but restored corticosterone to basal levels. As shown by Martin and colleagues, female rats did not display HFD-induced hyperglycemia as opposed to male rats [51], which may explain the lack of effects of ASH on the blood glucose of obese animals. These findings also indicate gender-related responses to diets and, potentially, gender differences in energy expenditure [51]. In summary, these outcomes indicate the potential of ASH to improve obesity-induced cognitive impairment. Future investigations should determine the specific ASH constituents responsible for its effects.

### 5.2. Adzuki Bean (Vigna angularis)

In East Asia, *Vigna angularis* (VA) is widely cultivated as an important food crop and as a medicinal plant with antipyretic, anti-inflammatory, and anti-edematous actions. Kitano-Okada et al. [52] showed that VA could effectively improve lipid profiles indicating its anti-hyperlipidemic and anti-obesity effects. The memory and cognitive enhancing effect of VA in an obese mouse model was recently examined by Choi and colleagues [40]. Mice were given HFD for 16 weeks and 4 weeks and were exposed to VA extract at doses of 100 and 200 mg/kg. In their study, they found that VA enhanced the spatial and recognition ability of obese mice as measured by the T-maze (*p* < 0.05), Morris water maze (*p* < 0.05), and novel object recognition tests (*p* < 0.05), respectively. VA also significantly decreased the body weight of obese mice. There are various saponins and flavonoids that may play a role in both the cognitive and weight loss effects of VA. Vitexin and isovitexin (an isomer of vitexin) may contribute to the improvement in cognition [40,53], given that these compounds improved scopolamine-induced memory impairment in animal models [54] and decreased the expression of AChE [55]. Other VA polyphenols may contribute to the anti-obesity effects of the plant and its effect on adipose tissues [56]. Given the remarkable effect of VA not only in decreasing body weight but also improving cognition in obese animals, there is a need to fully characterize the specific compounds mediating these effects and to purify them as potential treatments for obesity and associated cognitive impairment.

### 5.3. Dwarf Goat’s Beard (Aruncus dioicus var. kamtschaticus)

Substantial amounts of phenolics are contained within *Aruncus dioicus* var. *kamtschaticus*; hence, the interest among researchers is to study the anti-inflammatory, anti-diabetic, and anti-obesity activities of the plant. A recent study also sought to clarify the therapeutic potential of *Aruncus dioicus* in obesity-induced diabetes and cognitive dysfunction in C57BL/6 mice [41]. The study found that the ethyl acetate fraction from *Aruncus dioicus* var. *kamtschaticus* (EFAD; 20 and 40 mg/kg of body weight), given for 4 weeks, significantly improved working memory, spatial cognition, and short-term memory ability of HFD-fed obese mice as evaluated by the Y-maze (*p* < 0.05), Morris water maze (*p* < 0.05) and passive avoidance tests (*p* < 0.05), respectively. EFAD was found to improve HFD-induced oxidative stress, reduce AChE activity, increase ACh contents, and alleviate mitochondrial dysfunction in the brain tissue of obese mice. It also effectively reduced the body weight of HFD-fed obese mice and improved impaired glucose tolerance, which was attributed to the improvement of energy homeostasis through the inhibition of lipogenesis and enhancement of fatty acid oxidation in adipocytes. The anti-obesity and anti-diabetic effects of EFAD were linked to phenolics, such as dicaffeoyl glucose isomers, which were also suggested to have cognitive-enhancing actions [41]. Additionally, the presence of caffeic acid in *Aruncus dioicus*, which has an excellent α-glucosidase inhibitory effect [57], was associated with the improvement of energy metabolism in EFAD-treated obese mice and the extract’s anti-oxidative properties. More research is needed to verify the cognition-improving effects of the above-mentioned substances (and other phenolics) and the value of *Aruncus dioicus* var. *kamtschaticus* as a functional food substance used to enhance obesity-induced cognitive dysfunction and neurodegeneration.

### 5.4. Hardy Kiwi (Actinidia arguta)

The hardy kiwi (*Actinidia arguta*) is a widely grown perennial vine in northeastern Asia, known to contain vitamins, polyphenols, some minerals, amino acids, and fatty acids. Pharmacologically, the fruit extracts of the hardy kiwi have shown significant antioxidant and anti-inflammatory activities [58] and have been found to improve glucose tolerance in diabetic mice [59]. These properties have led Ha and colleagues [42] to investigate the efficacy of chloroform fraction of the *Actinidia arguata* (CFAA) to improve brain dysfunction in HFD-induced obese mice. Their study revealed that obese mice supplemented with the CFAA (40 mg/kg, for 4 weeks) displayed improvement in glucose tolerance and, importantly, working memory, short-term memory ability, and spatial cognitive function as assessed by the Y-maze (*p* < 0.05), passive avoidance (*p* < 0.05), and the Morris water maze tests (*p* < 0.05), respectively. CFAA supplementation also decreased brain expression of AChE, lowered levels of oxidative stress markers, and improved mitochondria activity. Additionally, CFAA improved HFD-induced abnormal insulin signaling and prevented neuronal apoptosis by suppressing the Janus Kinase (JNK) pathway. UPLC Q-TOF/MS chromatography was conducted to determine the major bioactive compounds in the CFAA revealing significant amounts of pentacyclic triterpenoids such as madecassic acid and asiatic acid, which have been previously shown to exert anti-diabetic effects. The direct involvement of these substances in improving cognitive dysfunction in obese animals seen in the study of Ha and colleagues requires further investigation.

### 5.5. Japanese Aster [Aster yomena (Kitam.) Honda]

*Aster yomena (Kitam.)* Honda (AY) is known for its anti-inflammatory, anti-cancer, and antioxidant activities, as well as its potential to promote weight loss and block abnormal AChE activity [60]. Kim and colleagues [43] investigated the potential of the ethyl acetate fraction from AY (EFAY) to ameliorate obesity-induced learning and memory impairment in mice. Mice exposed to HFD exhibited cognitive deficits, but treatment with EFAY (100 and 200 mg/kg for 4 weeks) improved their working memory and spatial cognitive functions as assessed by the T-maze (*p* < 0.05) and novel object recognition tests (*p* < 0.05), and the Morris water maze tests (*p* < 0.05), respectively. The authors suggested that the cognitive-enhancing effects of EFAY may be attributed to its ability to decrease brain expression of NF-κB, IL-1β, and inflammatory mediators inducible nitric oxide synthase (iNOS) and cyclooxygenase-2. Moreover, they also proposed that EFAY may improve brain insulin resistance by regulating the IRS-1/Akt pathway. Further studies are needed to elucidate the precise molecular mechanisms responsible for EFAY’s cognitive-enhancing effect. Active compounds such as apigenin, esculetin, and caffeic acid, which have been found to possess neuroprotective and/or antioxidative properties in other studies [61,62], warrant further investigation in this regard.

### 5.6. Mango Ginger (Curcuma amada)

*Curcuma amada roxb*. (Mango ginger) rhizome is a highly potent plant with anti-inflammatory, antihyperlipidemic, and antioxidant activities that may ameliorate obesity-induced cognitive impairment. In a previous study, Rao et al. [44] explored this possibility and found that supplementation of the acetone extract of *Curcuma amada* (CAAE; 300 mg/kg of body weight) in obese rats enhanced working memory and learning of these animals as evaluated by the Y-maze (*p* < 0.05) and pole climbing tests (*p* < 0.05). The authors attributed the effects of CAAE to the inhibition of AChE activity, increased synaptic levels of serotonin and dopamine in the brain, and prevention of HFSD-induced hippocampal lipid peroxidation and neurodegeneration. In addition to its memory-improving effects, CAAE prevented weight gain in obese rats, potentially by attenuating dyslipidemia and enhancing high-density lipoprotein (HDL) levels in the plasma. Several components of the extract may contribute to the above-described effects, including curcuminoids and terpenoids [44], which are known to have weight loss and neuroprotective activities. Further testing is necessary to evaluate the potential of CAAE in addressing both obesity and obesity-linked cognitive outcomes.

### 5.7. Mulberry Root-Bark (Mori radices cortex)

*Mori radices cortex*, the root bark of Moraceae species, exhibits diverse pharmacological activities such as anti-inflammatory, anti-diabetic, and anti-obesity effects [63,64]. This is due to its abundant bioactive compounds such as stilbene, flavonoids, and alkaloids. Additionally, it has radical scavenging, antioxidative and anti-apoptotic effects in neuronal cells exposed to high glucose levels [65]. In a recent study by You and colleagues [45], HFD-fed mice were administered dissolved *Mori cortex radices* extracts (MCR; 100 or 200 mg/kg/day of body weight) for 6 weeks. The treatment resulted in improved working memory of obese mice as measured by the Y-maze-task (*p* < 0.05). MCR suppressed weight gain, inhibited AChE expression, and the production of malondialdehyde (MDA), a marker of oxidative stress. Additionally, it reduced p-Tau expression and increased the Bcl-2/Bax ratio, which may confer additional neuroprotective effects. These effects were attributed to the extract’s prenylflavonoids, which include cyclomulberrin, morusin, sanggenonI, and kuwanonU [45]. Notably, MCR improved glucose tolerance and reversed HFD-induced downregulation of p-IRS, PI3K, p-Akt, and GLUT4 levels, indicating an ability to improve insulin signaling. Overall, these findings suggest that MCR and its bioactive constituents have promising therapeutic potential as neuroprotective agents and cognitive enhancers in the context of HFD-induced obesity. However, further studies are necessary to confirm these effects and determine the optimal therapeutic doses.

### 5.8. Olive (Olea europaea)

Olive leaf extract is rich in polyphenols, such as oleuropein and oleanolic acid, which are its major physiologically active compounds. Oleuropein has been shown to decrease inflammation, blood pressure, and cholesterol levels and to exert potent antioxidant effects in vivo and in vitro [66,67]. Meanwhile, oleanolic acid has been found to increase energy production or mitochondrial biogenesis in cells via agonism of bile-acid-activated transmembrane G protein-coupled receptor 5 (TGR5) [68]. Previous studies have shown that these compounds prevented obesity in HFD-fed animals [69]. Recent findings also suggest that they may improve cognitive function in physically inactive obese animals. Specifically, Mikami and colleagues [46] reported that supplementation with 1 g of olive leaf extracts per 1000 g of HFD improved the working memory of HFD-fed, obese mice as evaluated by the Y-maze test (*p* < 0.05). Remarkably, the treatment also reduced the body weight of obese mice and enhanced their endurance exercise capacity. The weight loss effect was attributed to increased lipolysis and agonism of TGR5 (by oleanolic acid), which increased β-oxidation and energy production while reducing fat accumulation. Meanwhile, cognitive enhancement was ascribed to the enhancement of mitochondrial function or antioxidant capacity and BDNF expression in the hippocampus. Notably, the olive leaf extract also ameliorated depression-like behaviors (*p* < 0.05) of obese mice, indicating that part of the cognition-improving effect of the extract can be explained by its antidepressant activity. However, given that olive leaf extracts contain other bioactive polyphenols, it is important to determine whether the effects observed in the study were solely produced by oleuropein and/or oleanolic acid or by other compounds. Future studies should also utilize other behavioral assays (e.g., Morris water maze and novel object recognition tests) to comprehensively evaluate the therapeutic potential of olive leaf extract and its components in obesity-induced cognitive impairment.

### 5.9. Pineapple (Ananas comosus)

The pineapple (*Ananas comosus*) is a rich source of nutrients and bioactive compounds, including bromelain and polyphenols. Previous studies have shown that solvent extracts from pineapple possess anti-obesity and anti-inflammatory properties [70]. Building upon these findings, Ajayi et al. [47] investigated the potential of pineapple peel extract (PEAC) to improve memory impairment induced by HFD in animals. The researchers found that obese rats orally treated with PEAC (200 mg/kg) demonstrated improved working and recognition memory as assessed by the Y-maze (*p* < 0.05) and novel object recognition tests (*p* < 0.05), respectively. PEAC was also observed to improve serum and brain antioxidant status by decreasing malondialdehyde and increasing glutathione (GSH) and catalase. Additionally, it reversed the HFD-induced increase in brain AChE activity and reduced inflammation by decreasing IL-6 levels. The authors also found significant improvement in the lipid profile (total cholesterol and LDL levels) and decreased risk of atherogenicity (increased HDL/LDL ratio) in HFD-fed rats given PEAC. Anxiolytic-like effects of PEAC were also observed in the elevated plus maze test. The composition of phenolic and flavonoid compounds responsible for the observed effects, particularly the cognitive improvement in obese rats, warrants further investigation. Overall, PEAC shows promise as a potential therapeutic agent for improving cognitive deficits and behavioral abnormalities associated with obesity, including anxiety.

## 6. Plant-Derived Substances Tested to Ameliorate Obesity-Induced Cognitive Impairment

Traditional medicine often relies on whole plants or mixtures of plants to ensure that synergistic interactions or multi-factorial effects between compounds in herbal extracts are not missed [12,71]. However, it is crucial to identify and characterize the biological and pharmacological effects of bioactive components to understand the properties, mechanisms of action, and potential toxic effects of plant extracts [12,72]. Separately isolating and testing each component allows for the identification of specific compounds that contribute to the overall effect of plant extracts and their combined action [73]. In the succeeding sections, we will discuss the various classes of plant-derived substances that have been evaluated for their cognitive-enhancing effects in animal models of obesity. Details pertaining to the phytochemicals, their effects, and the outcomes of studies investigating their mechanism of action (Table 2) are described below.

### 6.1. β-Glucan

β-glucan is a type of polysaccharide found in various sources, including yeast, bacteria, fungi, and cereals. It is widely sold as a dietary supplement, with health benefits such as cardiovascular disease prevention and immune system enhancement. A previous study investigated the cognitive effects of long-term β-glucan supplementation derived from oats [74] in obese mice fed a high-fat diet. Moreover, Pan et al. [92] also investigated the cognitive effects of β-glucan derived from a non-plant source (i.e., Shiitake mushroom, also known as *Lentinula edodes*). Shi et al. [74] showed that oat-derived β-glucan supplementation improved the object recognition and working memory of obese mice, as measured by novel object recognition object location (*p* < 0.05) and Y-maze tests (*p* < 0.05), respectively. They also reported that oat-derived β-glucan supplementation (7 g/100 g of daily food intake) reduced HFD-induced microglia activation and prevented synaptic puncta engulfment in the hippocampus [74], and decreased the upregulation of pro-inflammatory cytokine mRNA expression (TNF-α, IL-1β, and IL-6). Moreover, oat-derived β-glucan promoted hippocampal PTP1B-IRS-pAKT-pGSK3β-pTau signaling for synaptogenesis improved the synaptic ultrastructure and increased both pre- and postsynaptic protein levels compared to the HFD-treated group. Similarly, Pan et al. [92] found that *L. edodes*-derived β-glucan supplementation (~1.5 mg/day) had anti-inflammatory and synaptic plasticity effects in both the hippocampus and the PFC.

Aside from the above-mentioned central effects of β-glucan, the compound also significantly improved HFD-induced gut barrier dysfunction in obese mice. Both Shi et al. and Pan et al. reported that β-glucan supplementation increased the thickness of colonic mucus and levels of tight junction proteins occludin and zonula occludens-1 in the colon of obese mice. Moreover, while Shi et al. reported that β-glucan supplementation reduced bacterial endotoxin translocation; Pan et al. reported that *L. edodes* β-glucan prevented the accumulation of pro-inflammatory macrophage and decreased LPS expression. Lastly, Shi et al. reported that even short-term β-glucan supplementation (7 days) reversed HFD-induced alteration of gut microbiota by preventing a further decrease in bacteria belonging to the Bacteroidetes phylum and the further increase in bacteria in the Firmicutes phylum. This is an important finding indicating the efficacy of β-glucan in changing the Firmicutes/Bacteroidetes ratio—the proposed marker of obesity [93].

It is worth noting that both studies showed that long-term β-glucan supplementation significantly reduced the body weight of obese mice. There are several types of β-glucans due to their variable glycosidic linkages. Future studies should investigate which type of β-glucan is beneficial, as spatial arrangements of molecules play a crucial role in the biological activity of compounds. Overall, the above studies suggest a cognition-improving effect of β-glucan attributable to its multiple beneficial effects exerted along the gut microbiota-brain axis.

### 6.2. Caffeine

Caffeine is a common psychoactive substance found in beverages such as coffee, tea, and energy drinks that is often used to improve mental alertness. Moy and McNay [75] investigated the impact of caffeine treatment (20 mg/kg for 11 weeks, once per week) on cognitive deficits induced by obesity in mice. The investigators found that supplementing caffeine to the mice’s high-fat diet improved working memory as evaluated by the four-arm maze tests (*p* < 0.05). Additionally, they also reported elevated BDNF levels in the hippocampus of caffeine-treated mice. Caffeine-supplemented mice also showed significantly less weight gain than the control HFD group. The study also found that caffeine did not affect hippocampal metabolism or insulin signaling, as HFD did not induce diabetes nor impair hippocampal insulin signaling or metabolism in mice. Notably, the cognitive enhancing and weight-reducing effects of caffeine were more pronounced in HFD-fed animals, indicating a specific interaction of caffeine with HFD. Further studies are required to explore the cognitive and weight-reducing effects of caffeine, considering the numerous biological effects of caffeine, such as the antagonism of adenosine receptors and regulating blood supply [75].

### 6.3. Chlorogenic Acid

Chlorogenic acid (CGA; 5-O-caffeoylquinic acid) is a phenolic acid metabolite that is found in many plants, including coffee beans, tomatoes, potatoes, and eggplants, and has potential therapeutic value in the treatment of various diseases. Previous preclinical studies have shown that CGA may be beneficial in the treatment of metabolic syndrome, nonalcoholic fatty liver, and type 2 diabetes mellitus [94,95]. CGA has been found to increase the percentages of *Blautia* and *Coprococcus* in the *Firmicutes* phylum and improve the plasma short-chain fatty acid (SCFA) profile in the spontaneous mouse model of metabolic syndrome, the Tsumura Suzuki obese diabetes mice [96]. Some central effects of CGA include increasing synaptic transmission or inhibiting the abnormal transformation of hippocampal plasticity [97], attenuating cognitive deficits, and decreasing Aβ plaque deposition via disaggregation of Aβ [98]. All these data suggest that CGA might also improve obesity-induced cognitive in animal models. Indeed, Zhang et al. [76] found that CGA treatment (150 mg/kg of body weight for 14 weeks) alleviated working memory and spatial cognitive function of obese mice as measured by the Y-maze (*p* < 0.01) and Morris water maze (*p* < 0.01) tests. This improvement was accompanied by the preservation of hippocampal neuron microstructure and alleviation of synaptic dysfunction as measured by H and E staining and electron microscopy. Moreover, transcriptomic analysis of the hippocampus revealed that CGA treatment stimulated gene expression associated with neurodevelopment and synaptic signal transmission while reducing the expression of neuroinflammation-related genes. Analysis of fecal samples also showed that CGA treatment increased the community, abundance, and phylogenetic diversity of microbiota and the level of bacterial genera producing SCFAs. CGA also reduced the concentration of energy metabolism substrates and increased phosphorylcholine, a precursor of ACh. These results suggest that CGA produces multiple effects that may have synergized to improve the cognitive dysfunction of HFFD-fed obese mice. Taken together, CGA is a promising functional phytochemical that can be further evaluated as a treatment to prevent cognitive deficits caused by metabolic disorders, e.g., obesity.

### 6.4. Curcumin

Curcumin, a bioactive compound responsible for the yellow color of turmeric, has long been studied for its potential therapeutic effects. Sarker et al. [77] examined the effect of curcumin administration (1000 mg/kg for 12 weeks) on a preclinical model of midlife sedentary obesity, inflammation, and comorbid cognitive impairment. Their study was conducted on middle-aged (15 months old) mice given ad libitum (AL) feeding of a purified maintenance diet. They reported that both curcumin-treated (CURAL) and AL mice had higher body weight compared to the calorie-restricted (CR) group. Curcumin, however, did not decrease adiposity but increased food intake compared to the AL group. The Morris water maze tests conducted after 8 weeks of treatment did not show a difference among treatment groups in the spatial learning index, indicating a lack of effect of curcumin on hippocampal-dependent learning. The T maze tests, however, showed that both CURAL (*p* < 0.05) and CR (*p* < 0.05), but not the AL group, required fewer trials to reach the criterion in the reversal sessions of the active avoidance task, indicating improvement in cognitive flexibility, a fronto-cortical function. Notably, the CURAL group had lower levels of C-reactive protein (CRP), a marker of low-grade inflammation, compared with the AL obese mice, in line with previous reports on the anti-inflammatory effects of curcumin via inhibition of nuclear translocation of NF-κB of activated B cells [99]. The CURAL, but not CR group, showed improvement in redox state, indicating that curcumin improved cognition, in part, via its antioxidant activity. Follow-up studies using various doses of curcumin may further elucidate the dose-response effects of curcumin on cognitive function. In summary, this study suggests that curcumin may have cognitive-enhancing effects in an animal model of midlife sedentary obesity attributable to its anti-inflammatory and antioxidant properties.

### 6.5. Formononetin

Formononetin, an isoflavone compound extracted from *Trifolium pratense* L., has been shown to possess anti-obesity, anti-inflammation, and neuroprotective effects. In a study by Fu et al. [78], formononetin treatment (20 and 40 mg/kg for 10 weeks) improved the spatial cognitive function of obese mice as evaluated by the Morris water maze test (*p* < 0.05). The authors reported that formononetin reversed the Tau hyperphosphorylation in the hippocampus of obese mice and reduced IL-1β and TNF-α levels. Formononetin was also found to attenuate the pro-inflammatory NF-κB signaling while enhancing the anti-inflammatory Nrf-2/HO-1 signaling pathway. The compound upregulated the peroxisome proliferator-activated receptor-gamma coactivator-alpha (PGC-1α), a molecule believed to promote mitochondrial biogenesis and exert anti-oxidation and anti-inflammation effects, and the authors suggest its involvement in the observed effects of formononetin. Moreover, formononetin treatment decreased body weight, blood glucose, total cholesterol, and triglyceride levels in obese mice. Additional studies are required to fully establish the efficacy of formononetin in reducing obesity-induced cognitive dysfunction and to characterize the role of PGC-1α.

### 6.6. Huperzine A

Huperzine A is a compound derived from *Huperzia serrata*, also known as Chinese club moss, used in traditional Chinese medicine. Huperzine A is deemed beneficial for people with AD due to its AChE inhibition activity. Wang et al. [79] investigated the effect of Huperzine A (0.1 and 0.3 mg/kg/day for 3 months) to ameliorate cognitive impairment induced by diet and in homozygous leptin knockout obese mice. The results showed that intragastric low dose (0.1 mg/kg/day) Huperzine A treatment improved the short-term recognition ability and spatial memory of diet-induced obese mice, measured by the novel object recognition (*p* < 0.05) and Morris water maze tests (*p* < 0.05), respectively. In leptin knockout mice, Huperzine A treatment increased their travel distance time, indicating an improvement in exercise capacity. Altogether these results suggest that Huperzine A may acutely improve obesity-induced cognitive impairment and improve the exercise capacity of animals. However, the efficacy of Huperzine A may be dose and administration-dependent, thus, warranting further pharmacokinetic studies. Despite the apparent improvement in exercise capacity, Huperzine A did not significantly impact the weight and insulin sensitivity of animals. Nevertheless, Huperzine A treatment increased pAKT levels, lowered BACE1 levels, and decreased Aβ42 levels in the cortex of animals. Intriguingly, the study results showed elevated cortical insulin levels in Huperzine A-treated mice, suggesting insulin insensitivity despite the significantly elevated pAKT levels. This paradoxical result requires further investigation in future studies.

### 6.7. Iso-α-Acids

Isomerized hop extract contains iso-α-acids, such as trans-isocohumulone, cis-isocohumulone, trans-isohumulone, and cis-isohumulone. Previous studies suggest that iso-α-acids can prevent dyslipidemia and type 2 diabetes in a diet-induced obese rodent model [100,101], improve glucose metabolism and reduce body fat in a clinical trial [102] and prevent cognitive decline in and inflammation induced by amyloid β accumulation [103]. Ayabe et al. [80] further explored the potential of iso-α-acids (0.05% *w*/*w*, for 8 weeks) to suppress neuroinflammation and cognitive-impairment in male mice fed with HFD. They found that iso-α-acids improved object recognition and spatial memory of obese mice, as assessed by the novel object recognition (*p* < 0.01) and object location tests (*p* < 0.05), respectively. In the hippocampus, the treatment attenuated neuroinflammation by suppressing IL-1β, IL-6, and TNF-α levels, as well as lipid peroxidation. The hippocampal weight of HFD-fed animals is usually lighter compared to controls, indicating brain atrophy in this brain area. Supplementation of iso-α-acids attenuated HFD-induced atrophy. Surprisingly, Ayabe et al. found increased BDNF levels in the hippocampus of HFD-fed animals, which they suggested was a compensatory response to HFD-induced hippocampal atrophy. Iso-α-acids prevented the increase in the levels of BDNF. Iso-α-acids supplementation decreased the body weight of obese mice but did not affect their food intake. However, iso-α-acids seemed to exert protective effects on internal organs via the activation of peroxisome proliferator-activated receptor alpha (PPARα) and gamma (PPARγ). Because activation of PPARα and PPARγ also produces anti-inflammatory effects in AD models [104] and improves spatial learning of HFD-induced obese rats [105], part of the cognitive effects of iso-α-acids in HFD-fed obese mice may be ascribed to the activation of the above-mentioned receptors. Further investigations are required to establish the therapeutic potential of iso-α-acids in reducing the risk of cognitive impairment in obese individuals.

### 6.8. Isorhamnetin

Isorhamnetin (ISO) is one of the major flavonoids found in Sea buckthorn. In a follow-up study, Mulati et al. [81] investigated the effects of ISO (0.03% *w*/*w* and 0.6% *w*/*w* for 14 weeks) in cognitively impaired obese mice. The authors reported significant improvement in working, spatial and long-term memory in ISO-treated obese animals, as evaluated by the Y-maze (*p* < 0.01), novel object recognition (*p* < 0.01), and Morris water maze tests (*p* < 0.01), respectively. The cognitive improvement due to ISO treatment was attributed to several factors. ISO was found to enhance the CREB/BDNF pathway and promote the expression of nerve growth factor (NGF), neurotrophin-3 (NT-3), and neurotrophin-4 (NT-4). ISO also attenuated neuroinflammation by significantly reducing microglial activation, the expression of pro-inflammatory cytokines (e.g., TNFa and IL-6), and the p38 MAPK pathway. Notably, ISO also produced other beneficial metabolic effects, such as weight loss that may be linked with improved insulin sensitivity and normalizing serum lipids (e.g., LDL, TC, TG, and HDL). ISO is a promising compound that could improve not only obesity but also obesity-linked cognitive outcomes.

### 6.9. Luteolin

Luteolin is derived from *Reseda luteola*, commonly known as dyer’s rocket, a plant used for its natural yellow dye. Liu et al. [82] investigated the effects of luteolin supplementation (10 mg/kg for 20 weeks) on the cognitive function of HFD-fed obese mice. The study found that luteolin improved spatial memory and learning of obese mice as tested by the Morris Water maze test (*p* < 0.05) and a passive avoidance task (*p* < 0.01). The researchers also observed that luteolin improved antioxidant properties in both the hippocampus and the cortex by increasing superoxide dismutase (SOD) and GSH and decreasing the level of the oxidative marker malondialdehyde (MDA). Furthermore, luteolin reduced central proinflammatory cytokines such as TNF-a, IL-6, IL-1B, and NF-κB while also reducing MCP-1 and plasma resistin and increasing plasma adiponectin levels. These findings suggest that luteolin treatment may decrease global inflammation and improve insulin sensitivity. Additionally, luteolin treatment resulted in a significant increase in synaptic proteins, PSD95, and BDNF, which may improve synaptic resilience and plasticity. Finally, luteolin treatment was also associated with a reduction in body weight of obese mice, possibly due to decreased food intake and improved peripheral insulin sensitivity. Follow-up studies using various doses may further verify dose-response effects on the cognitive function of obese individuals.

### 6.10. Naringin

Naringin, a flavonoid glycoside, is one of the molecules responsible for CYP inhibition [106] and lends grapefruit its notorious bitter taste. It possesses numerous biological activities such as antioxidant, anti-inflammatory, and anti-apoptosis effects [107]. Several studies also reported the cognition-improving effect of naringin in various animal models [108,109]. Wang et al. [83] investigated the effects of naringin on cognitively-impaired obese mice. They found that 20 weeks of naringin treatment (100 mg/kg) improved the working memory and spatial learning of obese mice as tested by the novel object recognition test (*p* < 0.01) and the Morris Water maze tests (*p* < 0.01). Naringin supplementation also enhanced hippocampal insulin signaling and attenuated mitochondrial dysfunction in obese mice. The investigators observed increased AMPK activity in the hippocampus of naringin-treated obese mice suggesting a role of this pathway in both restorations of insulin signaling and mitochondrial dysfunction. It is worth noting that naringin supplementation reduced the body weight of HFD-fed obese mice. The decrease in body weight may be attributed to the improved peripheral insulin sensitivity as tested by glucose tolerance tests and serum insulin measurements. Naringin supplementation also reduces free fatty acids and total cholesterol levels, benefiting cardiovascular health. Naringin supplementation improved the cognitive and metabolic functions of obese mice but warranted safety and pharmacokinetic studies due to its effects on CYP enzymes.

### 6.11. Panax japonicus Saponins

*Panax japonicus* (PJ), the dried rhizome of *Panax japonicus C. A. Mey*., contains saponins with potent anti-inflammatory properties [110]. Yuan’s group previously showed that saponins from *Panax japonicus* (SPJ) attenuated HFD-induced fatty liver fibrosis [111] and reversed the cognitive decline of aged rats via inhibition of NLRP3 inflammasome activation. Based on these findings, they sought to investigate the cognitive-enhancing effect of SPJ in HFD-fed, obese mice [84]. They showed that oral administration of SPJ (15 mg/kg and 45 mg/kg for 16 weeks) improved spatial learning of obese mice as measured by the Morris water maze test (*p* < 0.01). Concomitantly, it prevented further body weight gain and improved glucose tolerance in obese animals. The morphological changes in hippocampal neurons due to HFD exposure were reversed by SPJ treatment in mice. SPJ treatment also attenuated neuroinflammation by decreasing iNOS, COX-2, and TNF-α expression in the cortex and hippocampus of HFD-fed mice and by preventing the activation of the NLRP3 inflammasome, an important neuroinflammation- and neurodegeneration-associated molecule. Furthermore, SPJ treatment reversed the HFD-induced decrease in AMPA receptor expression in the cortex and hippocampus. It increased the levels of phosphorylated CaMKII and CREB, the molecules that mediate the AMPA receptor signaling pathway. All these effects may further contribute to the memory-enhancing effect of SPJ. Interestingly, aside from improving cognition, SPJ also exerted antidepressant-like effects in obese mice. The effects of SPJ are multi-faceted necessitating further studies to fully determine its value in the treatment of cognitive impairment and other behavioral abnormalities (e.g., depression) due to HFD and obesity.

### 6.12. Purple Sweet Potato Color Anthocyanins

Anthocyanins have become a focus of research for their potential effects on neuronal activity and functions. Purple sweet potato color (PSPC) contains anthocyanins [109], and previous studies showed that PSPC exerts neuroprotective activities and activates the AMPK, which subsequently enhances autophagy, a critical process for neuronal functions and survival [85]. These findings indicate the potential of PSPC to protect against cognitive impairments due to HFD and obesity. In a study by Zhuang et al. [85], PSPC treatment (100 mg/kg + HFD; 20 weeks) significantly improved short-term memory and spatial cognition of obese mice as measured by the passive avoidance (*p* < 0.05) and Morris water maze tests (*p* < 0.01), respectively. It also reduced body weight (without affecting food intake) and improved peripheral insulin resistance in obese mice. Moreover, PSPC treatment enhanced autophagy by restoring the levels of autophagy-related proteins (e.g., p-ULK1, Beclin 1) and blocked oxidative stress by decreasing the levels of protein carbonyls, malondialdehyde, and ROS in the hippocampus of HFD-fed mice. Hippocampal BDNF expression and neuron survival were also increased in PSPC-treated obese mice, indicating neuroprotective effects. These improvements were mediated, at least in part, by the activation of AMPK because the treatment of metformin mimicked the effect of PSPC [112]. Further studies are warranted to understand further the mechanisms underlying the effect of PSPCs and anthocyanins and to establish the role of PSCPs in improving cognitive impairment due to HFD and obesity.

### 6.13. Rhein

Rhein is the active component of rhubarb, a hardy perennial in the buckwheat family (Polygonaceae). It has been reported to exert various biological effects, including decreasing body weight gain and fat accumulation in HFD-induced obese mice [113,114]. A study by Wang et al. [86] evaluated the potential effects of 98% rhein extract (120 mg/kg for 6 weeks) on the cognitive impairment of diet-induced obese mice. The results showed that rhein supplementation improved the working memory of obese mice assessed through the novel object recognition test (*p* < 0.05). Rhein may have also reduced neuroinflammation, as shown by the attenuation of both the NF-kB pathway and expression of pro-inflammatory cytokines in the perirhinal cortex in rhein-treated obese mice. In addition, rhein prevented gut dysbiosis, reduced expression of colonic pro-inflammatory cytokines, and reduced plasma LPS. The exact mechanism underlying rhein’s regulation of microbiota was not fully determined. The reduction in plasma LPS suggests minimal activation of the brain’s innate anti-inflammatory response, shown by decreased expression of toll-like receptor 4 (TLR4) in the perirhinal cortex. Finally, rhein treatment also resulted in body weight reduction in treated obese mice. Further studies are needed to verify the potential application of rhein in improving obesity-induced cognitive deficits.

### 6.14. Roxburgh’s Jewel Orchid Polysaccharides

A component of traditional Chinese medicine, *Anoectochilus roxburghii (Wall.) Lindl* contains polysaccharides (ARPs) with multiple pharmacological activities, namely, antioxidant, anti-inflammation, anti-hyperglycemia, and anti-hyperlipidemia [115,116]. ARPs also improved obesity-induced inflammation [117] and significantly prevented aging in a D-galactose-induced dementia model [118]. Furthermore, ARPs decreased the pH value in the cecum and increased its content of probiotic bifidobacteria [119]. Fu et al. [87] investigated whether dietary supplementation of ARPs can improve cognitive functions in mice induced by HFD exposure. Central effects of oral ARPS (1 and 3 mg/g *w*/*w* for 14 weeks) include improving spatial learning and working memory of mice measured by the Morris water maze (*p* < 0.01) and Y-maze tasks (*p* < 0.01), respectively. The treatment also attenuated HFD-induced neuroinflammation, decreased the phosphorylation levels of Tau protein in the hippocampus, and increased the level of BDNF. Peripherally, ARPs blocked the HFD-induced increase in plasma glucose, total cholesterol, and inflammatory factors and decreased the body weight of obese animals. ARPS also improved the relative abundance of several bacteria genera, including Parabacteroides, which may play regulatory roles in cognitive function. The upregulation of intestinal tight junction proteins, which indicate effectiveness in restoring the intestinal epithelial barrier, is another notable effect of ARPs. All these effects indicate the influence of ARPs on the gut-brain axis, thereby improving the cognitive impairment induced by obesity. It is possible that flora-metabolites also participated in the anti-inflammatory and neuroprotective effects of ARPs [87]. This possibility and the exact mechanism through which ARPs restore intestinal barrier integrity and ameliorate obesity-induced gut dysbiosis must be confirmed in future studies.

### 6.15. Sea-Buckthorn Flavonoids

Sea-buckthorn (*Hippophae rhamnoides*), a new berry fruit bush of the Elaeagnaceae genus, contains flavonoids that have been shown to prevent metabolic complications caused by diets such as dyslipidemia, obesity, and inflammation. Recently, it was reported that sea-buckthorn flavonoids (SFs) could also prevent memory impairment induced by high-fat and high-fructose diets (HFFD) [88]. Mulati et al. showed that SFs (0.06% and 0.31% *w*/*w*, for 14 weeks) improved working memory, spatial cognition, and object recognition of obese mice as evaluated by the Y-maze test (*p* < 0.01), Morris water maze (*p* < 0.01), and novel object recognition tests (*p* < 0.01), respectively. The brain effects of SFs include attenuation of HFFD-induced synaptic dysfunction and neuronal loss in the hippocampus and normalization of the ERK/CREB/BDNF and IRS-1/AKT signaling pathways. Sea-buckthorn also produced an anti-inflammatory effect via the inactivation of the NF-κB signaling and expression of downstream inflammatory mediators. Peripherally, SFs blocked HFFD-induced glucose intolerance increase and insulin sensitivity, as well improved lipid profiles of obese mice. SFs also reduced the body weight of obese mice. The flavonoids within Sea-buckthorn, such as quercetin, isorhamine, and catechin, may have produced the above-mentioned effects, individually or in combination with other flavonoids. In particular, the major SF quercetin may mediate sea-buckthorn’s neuroprotective and anti-inflammatory effects, as well as the restoration of insulin-dependent signaling [88]. Catechins and isorhamnetin may also play additional neuroprotective roles and subsequently improve the cognition of HFFD-fed obese mice [88]. Further work is needed to fully ascertain the therapeutic effects of individual SFs and their complementary actions in improving the cognitive outcomes of obesity.

### 6.16. Silymarin

Silymarin is a polyphenolic flavonoid extracted from the seeds of *Silybum marianum* or milk thistle, which is a free radical scavenger used to treat hepatic disorders such as cirrhosis [120,121]. Neha et al. [89] investigated the effects of silymarin supplementation (100 and 200 mg/kg for 15 days) on the cognitive function of HFD-fed obese mice. The results of the study showed the silymarin supplementation significantly improved the spatial memory of obese mice as measured by the Morris Water maze test (*p* < 0.05). The enhanced cognitive performance may be attributed to the decreased activity of AchE, which allowed for improved synaptic signaling. Furthermore, silymarin supplementation resulted in increased brain GSH levels and attenuated myeloperoxidase (MPO) activity, which is consistent with the reduction in oxidative stress markers shown by the quantification of thiobarbituric acid reactive species (TBARS). Additionally, the study found that silymarin supplementation normalized nitric oxide levels in the brain, as evidenced by a reduced brain nitrite-to-nitrate ratio. This is significant because nitric oxide, while healthy for the brain at normal physiological levels, can contribute to neurological conditions such as AD when increased. Lastly, the investigators also observed that silymarin significantly decreased HFD-induced weight gain and decreased total cholesterol levels. Despite the promising results, the exact mechanisms of and molecules responsible for these cognitive and metabolic effects of silymarin require further investigation.

### 6.17. Tea Saponins

Tea has long been known to possess medicinal properties, including anti-inflammatory and anti-obesity effects [122]. Previous studies have shown that tea can also improve gut microbiota [123,124]. Tea extracts contain two major bioactive compounds, phenolics and saponins. There is growing interest in understanding the impact of dietary polyphenols on the intestinal microbiota and the effects of this interaction on the gut-brain axis. Wang et al. [90] investigated the effects of dietary tea saponin on the alteration of gut microbiota and cognitive decline in diet-induced obese mice. They found that tea saponin treatment (0.5% mixed in diet for 6 weeks) prevented the HFD-induced recognition memory deficit as measured by the novel object recognition test (*p* < 0.05). The hippocampi of tea saponin-treated obese animals expressed higher levels of BNDF compared to controls, and neuroinflammation and gliosis were also attenuated in this brain region. Interestingly, tea saponin treatment produced weight loss and improved glucose tolerance in obese mice. In addition to these effects, it also decreased the HFD-induced endotoxemia and pro-inflammatory M1 macrophage accumulation in the colon. Teasaponins also increased the *Bacteroides–Prevotella* spp. and decreased *Lactobacillus* spp. belonging to Bacteroidetes and Firmicutes, respectively. This is indeed a notable change, given that in obesity, there is a marked increase in the ratio between Firmicutes and Bacteroidetes [125]. Further studies using 16S rRNA sequencing or metagenomic sequencing studies can help verify these remarkable effects of tea saponins. It is also important to clarify how improvement in gut microbiota can lead to cognitive benefits. The above study results indicate that tea saponins could improve the microbiota and induce many beneficial effects, such as weight loss and attenuation of obesity-induced cognitive dysfunction.

### 6.18. Xanthohumol Derivatives

Xanthohumol (XN) is the principal prenylated flavonoid found in hops (*Humulus lupulus*), which has been shown to have anti-obesity effects in mice and rats [91,126]. Additionally, XN has been found to improve metabolic aberrations in metabolic syndrome [91]. Although it has an excellent safety profile with no detectable toxicity at doses up to at least 1000 mg/kg in mice [127], concerns have been raised about its use in dietary supplements due to the formation of a very potent phytoestrogen, the 8-prenylnaringenin (8-PN) [128]. To address this issue, XN derivatives have been developed that cannot be metabolized to 8-PN; and their roles in mitigating cognitive impairments associated with obesity have been investigated. Two such derivatives, DXN and tetrahydro-XN (TXN), were found to have negligible affinity for estrogen receptors α and β and were not metabolically converted into 8-PN [129]. Treatment with XN (*p* < 0.05), DXN (*p* < 0.05), or TXN (30 mg/kg for 13 weeks) (*p* < 0.05) ameliorated HFD-induced deficits in spatial learning and memory of obese mice measured by the Morris water maze tests. Given that these compounds improved the glucose tolerance of mice, part of their cognitive enhancing effects were attributed to the improvement of insulin signaling. Notably, only TXN was found to reduce body mass gain, feed efficiency, and fasting plasma glucose levels, which may underlie increased energy expenditure and weight loss in TXN-treated obese mice. DXN and TXN also improved markers of peripheral metabolism in HFD-fed mice without inducing liver toxicity. These results suggest that the XN derivatives show promise as effective treatments for reducing the neuro-metabolic consequences of HFD-induced obesity.

## 7. Concluding Remarks

Although more research is needed in the area, accumulating evidence suggests that obesity adversely affects cognitive functions, including executive functions, learning, and memory. New research has emerged, further clarifying the pathophysiological mechanisms of obesity-induced cognitive impairment, including recent studies that have examined the therapeutic potential of plants (Table 1) and isolated compounds (Table 2). It is worth noting that our search was limited to a single database, which may have resulted in the exclusion of pertinent plants and isolated compounds. Nevertheless, the treatments examined in this review demonstrated significant efficacy in improving the cognitive functions of obese animals. Further studies are needed to elucidate the precise mechanisms by which the aforementioned plants and isolated compounds exert their effects. Additionally, exploring whether plant extracts are superior to isolated compounds in improving cognitive outcomes in obesity would be a valuable avenue for future research. Conducting a meta-analysis to evaluate and compare their effects could provide a more comprehensive understanding of this issue.

Recent studies have also shown the influence of plants and specific isolates on the gut microbiota. Future studies should examine the interactions between plants and plant-derived compounds and microbiota and how they influence the gut-brain axis to affect food intake and/or improve cognition. Medicinal plants are assumed to be safer than synthetic drugs. However, safety and toxicity assessments are still required as they may have metabolites that might be toxic or produce adverse side effects (e.g., 8-8PN).

Regarding preclinical testing, the behavioral assays used in the above studies to evaluate cognition are helpful but are simplistic and do not fully measure complex cognitive functions in humans. There is a need to use other behavioral assays in future studies that consider various aspects of cognition and behavior to improve the validity, reliability, and reproducibility of outcomes [130]. Clinical studies are also warranted to fully establish the therapeutic roles of natural dietary, plants, and isolated compounds in treating obesity-induced cognitive impairment. Notably, there is variability in diets introduced to produce obesity in animals. The diets used should fully recapitulate the nutritionally varied, energy-dense, and highly palatable grocery store-purchased foods that drive the current global obesity pandemic [131]. Moreover, there are sex differences in adiposity and inflammatory response, necessitating the inclusion of female subjects in preclinical testing [6]. This is also critical given that therapeutic targets and treatments may present variations and should, therefore, be tested in both male and female subjects [6].

Ideally, treatments should not just improve cognitive dysfunction but also promote weight loss [11]. It is noteworthy that several plants and plant-derived compounds included in this review have demonstrated the potential to induce both weight loss and improvements in cognition. Therefore, it is essential to explore the relationship between these two factors in treatments that yield simultaneous benefits in weight loss and cognitive enhancement. Further investigation is needed to determine whether cognitive improvement is a consequence of weight loss or vice versa. Moreover, some plant extracts and components from plants may have a therapeutic value in improving cognitive and other mental health outcomes (e.g., anxiety and depression) of obesity and/or poor diets. Investigating the potential synergistic effects on cognition, weight loss, and mental health outcomes through combined treatments of natural products, plants and plant-derived substances, synthetic drugs, and lifestyle interventions like exercise would be a valuable avenue for future research.

In conclusion, the findings from studies included in this review suggest that obesity-induced cognitive impairment can be ameliorated using plants and plant-derived sources. Further studies are needed to firmly establish the clinical efficacy of plants and plant-derived substances to treat cognitive impairment due to poor diets or obesity.

## Figures and Tables

**Table 1:** Plants with cognition-improving effect in diet-induced obese animal models.

Plant	Treatment Dose; Duration	Central Effects	Other Effects	Obesity Model; Strain, Species; Gender	Reference
Ashwagandha (*Withania somnifera*)	Dry leaf powder (1 mg/g of BW) + HFD; 12 weeks	neuroprotection; enhancement of synaptic plasticity and cell survival	normalized corticosterone levels; improved locomotor coordination	HFD (30% fat by weight); Wistar albino rats; Female	[39]
Adzuki bean (*Vigna angularis)*	Ethanol extract (VA 100; 200 mg/kg of BW) + HFD; 4 weeks	not investigated	weight loss	HFD (60% fat); C57BL/6J mice; Male	[40]
Dwarf Goat’s Beard (*Aruncus dioicus* var. *kamtschaticus*)	Ethyl acetate fraction (EFAD 20; 40 mg/kg of BW); +HFD 4 weeks	reduction of oxidative stress, improvement of impaired cholinergic system and mitochondrial dysfunction, etc.	weight loss; improved impaired glucose tolerance	HFD (20 kcal%/g fat); C57BL/6 mice; Male	[41]
Hardy kiwi (*Actinidia arguta*)	Chloroform fraction (20 and 40 mg/kg); 4 weeks	improved impaired cholinergic, antioxidant system, and mitochondria functions; brain insulin signaling	improved glucose tolerance	HFD (20 kcal%/g, carbohydrate 20 kcal%/g and fat 60 kcal%/g, 5.24 kcal/g); C57BL/6 mice; Male	[42]
Japanese aster [*Aster yomena (Kitam.) Honda*]	Ethyl acetate fraction (EFAY 100 mg/kg; 200 mg/kg) + HFD; 4 weeks	decreased neuroinflammation; ameliorated insulin resistance	not investigated	HFD (60% fat); C57BL/6J mice; Male	[43]
Mango ginger *(Curcuma amada*)	Acetone extract (CAAE 100; 300 mg/kg of BW) + HFHS diet; 3 weeks	reduction in AchE and oxidative stress markers, improvement of impaired cholinergic system; increased dopamine and serotonin levels; neuroprotection	weight loss; decreased liver and serum lipid levels; increased HDLs	HFHSD (3 mL of ghee and 1 mL of coconut oil, and 25% of fructose); Wistar albino rats; Male	[44]
Mulberry root bark (*Mori radicis cortex)*	Dissolved extracts (100 or 200 mg/kg/day of BW); 6 weeks	decreased AChE expression; reduction of oxidative stress; inhibition of p-Tau expression; neuroprotection	weight loss; inhibited disruptions of lipid metabolic markers; lowered blood glucose spikes	HFD (60% kcal fat); C57BL/6 mice; Male	[45]
Olive (*Olea europaea*)	Ethanol/Water extract of olive leaves OLEAVITA (1 g olive leaf extract per 1000 g of HFD); 10 weeks	improvement of mitochondrial function and antioxidant capacity; increased BDNFexpression	weight loss; enhanced mitochondrial muscle mass and endurance exercise capacity; antidepressant-like effect	HFD (protein, 17.8 g; fat, 20.0 g; carbohydrate 49.0 g; calorie, 480.8 kcal) + 200 g butter) C57BL/6J mice; Male	[46]
Pineapple (*Ananas comosus*)	Methanol extract of peel (PEAC 200 mg/kg of BW); 3 weeks	increasing antioxidant capacity; decreased brain AChE levels; reduction of neuroinflammation	anxiolytic-like effect; improved lipid profile and decreased risk of atherogenicity	HFD (44% animal fat and 0.3% methionine); Wistar rats; Male	[47]

Abbreviations: BW: body weight; HFD: high-fat diet; HFHSD: high-fat high-sucrose diet; HDL: high-density lipoprotein; AChE: acetylcholinesterase.

**Table 2:** Plant-derived compounds with cognition-improving effect in diet-induced obese animal models.

Isolated Compounds	Source(s)	Treatment Mode; Dose; Duration	Central Effects	Other Effects	Obesity Model; Strain; Species; Gender	Ref.
β-glucan	Oats	Oral β-glucan from oats (7 g/100 g of daily food intake) + HFD; 15 weeks and short-term for 1 week	decreased microglial activation and inflammation; promoted synaptogenesis; improved insulin signaling; inhibited Tau phosphorylation; improved synaptic plasticity	weight loss (long-term treatment); reversed gut barrier dysfunction; increased thickness of colonic mucus; increased level of tight junction proteins; ameliorated altered microbiota	HFFD (55% by energy; 5% fiber by weight); C57BL/6J mice; Male	[74]
Caffeine	Coffee, tea	Intraperitoneal caffeine; 20 mg/kg; once weekly; 11 weeks	reversed decrease in BDNF levels	weight loss; decreased plasma insulin levels	HFD (31.8% of energy as fat); SD rats; Male	[75]
Chlorogenic acid	Various plants	Oral chlorogenic acid; (150 mg/kg/day) + HFFD; 14 weeks	ameliorated hippocampal structural damage and synaptic dysfunction; decreased inflammation; improved cholinergic synapse and calcium signaling pathway	weight loss; reduced insulin resistance and lipid profile; increased gut microbiota diversity and bacteria producing SCFA; significant decrease in TG, TC, LDL, and significantly increased HDLs	HFHS (45% kcal from fat, 10% fructose in drinking water) C57BL/6J mice; Male	[76]
Curcumin	Turmeric (*Curcuma longa*)	Oral curcumin ad libitum; 1000 mg/kg diet; 12 weeks	anti-inflammatory or antioxidant actions	no weight loss effect	Ad libitum standard diet (4.1% energy as fat); C57BL/6 mice; Male	[77]
Formononetin	Red clover (*Trifolium pratense*)	Intragastric (20,40 mg/kg); 10 weeks	Decreased Tau hyperphosphorylation; reduced cytokines	weight loss	HFD (10% lard oil, 1% cholesterol, 0.2% cholate; 5% sucrose); ICR mice; Male	[78]
Huperzine A	Chinese club moss (*Huperzia serrata*)	Intragastric or oral Hup A (0.1 mg/kg/day and 0.3 mg/kg/day); 12 weeks	increased insulin and AKT activity; decreased beta-secretase expression	no weight loss effect	HFD (60% energy from fat, 20% from protein, and 20% from carbohydrates); C57 BL/6 mice; Male	[79]
Iso-α-acids	Hops (*Humulus lupulus*)	Oral iso-α-acids group; daily 0.05% (*w*/*w*) + HFD; 8 weeks	attenuated neuroinflammation and lipid peroxidation; prevented hippocampal atrophy	weight loss; decreased epididymal fat and plasma triglyceride levels	HFD (60 kcal% from fat); C57BL/6 J mice; Male	[80]
Isorhamnetin	Sea-buckthorn (*Elaeagnaceae* genus)	Oral daily isorhamnetin (0.03% *w*/*w* and 0.06% *w*/*w*) in HFFD; 14 weeks	inhibited microglial overactivation and neuroinflammation; increased activity of neurotrophic factors	weight loss; improved serum and liver lipids: TC, TG, LDL, HDL	HFHFD (45% kcal from fat, 10% kcal from fructose water); C57BL/6J; Male	[81]
Luteolin	Dyer’s rocket (*Reseda luteola*)	Oral daily; 10 mg/kg in HFD; 20 weeks	decreased neuroinflammation, oxidative stress, and neuronal insulin resistance; increased BDNF levels, synapsin U and PSD-95	weight loss; restored blood adipokines to normal level;	HFD (Energy as 15% protein, 43% carbohydrate, and 42% fat); C57BL/6J mice; Male	[82]
Naringin	Grapefruit (*Citrus × paradisi*)	Daily oral naringin; 100 mg/kg + HFD; 20 weeks	ameliorated mitochondrial dysfunction, improved insulin signaling pathway, AMPK	weight loss; restored abnormal glucose, fatty acid, and cholesterol metabolism	HFD (unknown composition); C57BL/6J mice; Male	[83]
*Panax japonicus* saponins	Japanese ginseng (*Panax japonicus*)	Oral daily saponins; 15 mg/kg and 45 mg/kg; 16 weeks	decreased neurodegeneration and neuroinflammation; upregulated AMPA receptors signaling pathway	weight loss; antidepressant	HFD (60% total calories from fat); Balb/c mice; Male	[84]
Purple sweet potato color anthocyanins	Sweet potato (*Ipomoea batatas*)	Oral daily PSPC; 100 mg/kg + HFD; 20 weeks	enhanced autophagy, decreased levels of ROS; improved BDNF levels	weight loss; ameliorated peripheral insulin resistance	HFD (60% calories from fat); ICR mice; Male	[85]
Rhein	Rhubarb (*Rheum rhabarbarum*)	Daily oral rhein; 120 mg/kg + HFD; 6 weeks	improved BDNF levels; decreased neuroinflammation	weight loss; inhibited the increase in plasma LPS level and the proinflammatory macrophage accumulation in the colon and alteration of microbiota, improved glucose tolerance	HFD (60% fat by calories); C57BL/6J mice; Male	[86]
Roxburgh’s jewel orchid polysaccharides	Roxburgh’s Jewel Orchid [*Anoectochilus roxburghii (Wall.) Lindl.*]	Oral daily ARPs; (1 mg/g and 3 mg/g (*w*/*w*) + HFD; 14 weeks	decreased Tau phosphorylation; and neuroinflammation	weight loss; decreased plasma glucose, total cholesterol, inflammation; restored intestinal epithelial barrier; decreased abundance of Parabacteriodes	HFD (60% kcal from fat); C57BL/6J mice; Male	[87]
Sea-buckthorn flavonoids	Sea-buckthorn (*Elaeagnaceae genus*)	Oral daily Sea Buckthorn flavonoid; 0.06% and 0.31% *w*/*w* mixed in HFFD; 14 weeks	alleviated the synaptic damages, reduced neuroinflammation, normalized insulin signaling, increased neurotrophic growth factors levels	weight loss; reversed glucose intolerance increase and insulin sensitivity loss; significantly decreased TG, TC, LDL, significantly increased HDLs	HFHFD (45% kcal from fat, 10% fructose in drinking water); C57BL/6J mice; Male	[88]
Silymarin	Milk thistle (*Silybum marianum*)	Oral daily silymarin; 100 mg/kg and 200 mg/kg + HFD; 15 days	reversed increase in AChE activity; decreased oxidative stress; reduced nitrate/nitrite levels and myeloperoxidase activity; decreased brain neutrophil infiltration and Aβ burden	weight loss; lowered serum cholesterol level;	Cholesterol-rich diet; HFD (310 g/1000 g of total diet from fat); Swiss Albino mice; Male and Female	[89]
Tea saponins	Tea plant	Oral daily tea saponins; 0.5% mixed in HFD; 6 weeks	suppressed neuroinflammation; reduced microglia and astrocyte accumulation; raised BDNF levels	weight loss; reversed alteration of gut microbiota and systemic inflammation; reduced M1 macrophage accumulation in the colon;	HFD (60% energy from fat); C57BL/6J mice; Male	[90]
Xanthohumol Derivatives	Hops (*Humulus lupulus*)	Oral daily xanthohumol derivatives; 30 mg/kg; 13 weeks	not investigated	weight loss (TXN); improvement of impaired glucose tolerance; decreased HOMA-IR and plasma leptin	HFD (60% fat; 20% carbohydrate; 20% protein); C57BL/6J mice; Male	[91]

Abbreviations: HFD: high-fat diet; HDL: high-density lipoprotein; AChE: acetylcholinesterase; BDNF: brain-derived neurotrophic factor; ROS: reactive oxygen species; AKT: serine/threonine kinase; PSD-95: postsynaptic density-95; AMPK: AMP-activated protein kinase.

## Data Availability

Not applicable.
